# Simultaneous Detection of Eight Dairy-Derived Components Using Double-Tube Multiplex qPCR Based TaqMan Probe

**DOI:** 10.3390/foods13203213

**Published:** 2024-10-10

**Authors:** Yingying Su, Lu Meng, Jiaqi Wang, Yankun Zhao, Nan Zheng

**Affiliations:** 1 State Key Laboratory of Animal Nutrition and Feeding, Institute of Animal Sciences, Chinese Academy of Agricultural Sciences, Haidian District, Beijing 100193, China; hnndsuying@163.com (Y.S.); menglu@cass.cn (L.M.); jiaqiwang@vip.163.com (J.W.); 2 Institute of Quality Standards and Testing Technology for Agro-Products, Xinjiang Academy of Agricultural Sciences, Urumqi 830091, China; yankunzhao90@163.com

**Keywords:** double-tube and multiplex qPCR, TaqMan probes, milk and dairy products, adulteration, PCR efficiency, limit of detection

## Abstract

The authentication of milk and dairy products has great significance for food fraud. The present investigation entailed the development of a novel method that amalgamates the double-tube approach with multiplex real-time polymerase chain reaction (PCR) technology, incorporating TaqMan probes, to facilitate the high-throughput screening and detection of animal-derived constituents within milk and dairy products. Eight dairy-derived animal-specific primers and probes were designed for the mitochondrial *cytochrome b* (*Cytb*) gene of eight dairy products, including cow, buffalo, yak, goat, sheep, horse, donkey, and camel. Through the developed double-tube detection assays, the above eight targets could be simultaneously identified with a detection limit of 0.00128–0.0064 ng/μL. The multiplex qPCR assay was effectively validated using simulated adulterated samples with different mixing ratios and demonstrated a detection limit of 0.1%. Upon analysis of 54 commercially available dairy products, a mislabeling rate of 33% was revealed. This method affords an efficacious means of detecting dairy product ingredients, thereby offering robust technical backing for market oversight and regulatory enforcement of milk and dairy products.

## 1. Introduction

The issue of identifying the animal species origin of milk and dairy products has garnered growing interest, particularly in relation to traceability, allergic reactions, and the provision of precise consumer information. Milk is an excellent and highly nutritious dietary component that is abundant in protein, essential fatty acids, vitamins, calcium, and potassium, among other minerals [1]. Non-cow dairy variants, including milk obtained from species such as camel, donkey, horse, yak, buffalo, sheep, and goat, have become increasingly popular among consumers because of their medicinal values, good edibility, and compositions closely resembling the profile of human milk [2,3,4,5]. However, influenced by yield, rarity, and nutritional value, the prices of specialty dairy products are relatively high, which has led to some deliberate adulteration, mainly by adding cheaper milk to specialty milk to make illegal profits [6,7]. Adulterated dairy products violate consumer rights but also pose health risks, including allergies [8,9]. Hence, strengthening the supervision of dairy products and combating adulteration are essential to uphold fair trade [10].

Several analytical techniques have been established for the purpose of identifying animal species within dairy products, predominantly relying on the protein and fat constituents, along with molecular biological examinations that are centered on DNA molecules. Among them, DNA sequences are a popular choice for species specificity and higher chemical and thermal stability [11]. At present, the nucleic acid detection methods widely used nationally and internationally include traditional PCR-gel electrophoresis [12], PCR-restriction fragment length polymorphism (RFLP) analysis [13], DNA barcoding, loop-mediated isothermal amplification (LAMP) [14], real-time quantitative PCR (qPCR) [15], and droplet digital (dd) PCR [16]. Among them, qPCR technology based on DNA and fluorescent labeling exhibits greater timeliness, sensitivity, and repeatability [17]. Fajardo et al. [18] used qPCR technology to differentiate red deer, fallow deer, and roe deer in meat mixtures. Similarly, qPCR has been successfully used to identify cow and buffalo species in milk from China, India, and Pakistan [19]. Therefore, the PCR method has the ability to distinguish closely related species and even different varieties of the same species [20]. Researchers increasingly have developed qPCR techniques to detect and identify animal-derived components, especially using TaqMan-based techniques, which can improve specificity, sensitivity, and reproducibility. Hossain et al. [21] demonstrated that TaqMan probe-based multiplex qPCR technology can identify ingredients from cattle, buffalo, and porcine materials in the food chain, thereby preventing unfair competition in the market environment. Guo et al. [22] observed that the triplex qPCR assay could effectively identify bovine and horse DNA in milk and dairy products based on TaqMan probes.

The identification of target genes suitable for amplification is another important consideration in qPCR methods, as DNA degradation during food processing may limit the availability of DNA fragments of sufficient length for qPCR analysis, thereby increasing the possibility of cross-reactivity with other animal species [20]. Dooley et al. [23] and Soares et al. [24] developed detection methods for identifying pork species around the mitochondrial *cytochrome b* (*Cytb*) gene fragment. In addition, many investigations have focused on two or three dairy-derived components, and fewer studies have investigated multiplex qPCR techniques for the simultaneous detection and quantification of animal-derived components in eight milk animal species [22,25,26]. Considering the growing consumption of characteristic dairy products, the development of high-throughput and sensitive assays to detect adulteration from characteristic milk sources are essential to protect consumer health and reduce economic losses. In this study, we established a double-tube and duplex real-time PCR assay based on TaqMan probes, and used a four-channel fluorescence qPCR instrument to detect eight milk animal-derived ingredients. The multiplex qPCR technique enables the simultaneous identification of target genes from eight distinct species across two PCR systems, thereby enhancing the efficiency of detection. The development of this approach offers an effective detection methodology and technical backing for the authentication of distinct dairy products.

## 2. Materials and Methods

### 2.1. Sample Collection

Raw milk samples from various animal sources were collected from different animal farms across the country. These included cow, buffalo, yak, sheep, goat, horse, donkey, and camel. Commercial dairy products from buffalo, yak, sheep, goat, horse, donkey, and camel were purchased from online e-commerce platforms to evaluate the utility of multiplex qPCR on processed samples. Furthermore, five species of plant materials, including soybean, corn, wheat, sweet potatoes, and rice were purchased from a local supermarket in Beijing as negative control samples. All samples were stored at −20 °C before analysis.

### 2.2. DNA Extraction

The milk powder samples were first prepared into reconstituted milk at a ratio of 1:8, and then pretreated with raw milk and liquid dairy products. In the pretreatment of raw milk and commercial liquid milk samples, the method for sediment collection and degreasing as outlined by Liao et al. was strictly followed [27]. Milk samples of 10 mL were subjected to centrifugation at a rate of 6000× *g* for a duration of 10 min at 4 °C in order to separate the fat and the majority of the supernatant. The pellet was subjected to washing procedures three times using 600 μL of phosphate-buffered saline (PBS) buffer, and subsequently transferred to a 1.5 mL centrifuge tube. It was then centrifuged at a speed of 6000× *g* for a period of 5 min. Following centrifugation, the pellet was finally resuspended in 200 μL of PBS. Subsequently, DNA extraction from the precipitation was carried out using a magnetic blood genomic DNA kit (Tiangen Biotechnology, Beijing, China) following the manufacturer’s recommendations. The following method was employed: 20 μL of proteinase K and 300 μL of lysate were added to the aforementioned centrifuge tube. The tube was incubated at 65 °C for 15 min to allow for protein digestion, cell lysis, and inactivation of intracellular nucleases. Following magnetic separation, the magnetic beads were thoroughly washed with 700 μL of buffer and 700 μL of rinse solution to remove impurities such as proteins, and nucleic acids were purified. Subsequently, DNA was extracted from the beads using 80 μL of elution buffer and transferred to a new 1.5 mL centrifuge tube. Control samples were processed utilizing the EasyPure Food and Fodder Security Genomic DNA kit (TransGen Bio, Beijing, China). The extracted genomic DNA concentration was determined by a Thermo NanoDrop 2000 spectrophotometer to ensure compliance with the requirements of qPCR detection. The isolated and purified DNA was stored at −20 °C for subsequent use.

### 2.3. Primers and Probes Design

All primers and probes were designed targeting the mitochondrial *Cytb* gene. Mitochondrial DNA sequences were downloaded from the GenBank database with the following references: cow (*Bos taurus*, GenBank No. NC_006853.1), buffalo (*Bubalus bubalis*, GenBank No. NC_006295.1), yak (*Bos grunniens*, GenBank No. NC_025563.1), goat (*Capra hircus*, GenBank No. NC_005044.2), sheep (*Ovis aries*, GenBank No. NC_001941.1), horse (*Equus caballus*, GenBank, No. NC_001640.1), donkey (*Equus asinus*, GenBank No. NC_001788.1), and camel (*Camelus bactrianus*, GenBank No. NC_009629.2).

Primer sequences, shown in Supplementary Materials, were aligned and compared by using MegAlign 7.1 software to identify conserved and variable regions. The PCR primers were designed in the conserved regions of genes using Primer 5.0 software, and their species specificity was assessed using NCBI primer-blast. The 5′ end of the probe was modified by adding various fluorescent reporter molecules such as 6-carboxyfluorescein (FAM), melanin extender (hexachlorofluorescein, HEX), sulforhodamine acid chloride (Texas Red), and anthocyanin fluorescent dye (Cyanine5, CY5).

In this study, multiplex qPCR was performed simultaneously in two tubes. All primers and probes were synthesized by Sangon Biotech (Shanghai, China) and purified by high-performance liquid chromatography (HPLC). The sequences of primers and probes are shown in Table 1.

### 2.4. Internal Positive Control (IPC)

In order to eliminate false negative results in PCR reactions, a recombinant IPC was designed and synthesized for the amplification system. The target DNA sequences of cow, buffalo, yak, goat, sheep, horse, donkey, and camel were synthesized and integrated into the *E. coli* pUC57 vector to obtain a standard DNA plasmid with multiple targets. Sequencing verification showed that a single copy of the expected sequence was inserted into each species without deletion or insertion mutation.

### 2.5. Specific Testing of Primers and Probes

In order to evaluate the specificity of primers and probes, genomic DNA of target species, IPCs, and negative control (soybean, corn, wheat, sweet potato, and rice) DNA were selected as reaction templates. The templates were subjected to a single-plex qPCR assay using primers and probes, and ddH_2_O was set as the blank control during the test process. The assay was performed in 20 μL reaction volumes containing 10 μL of 2× TaqMan Fast qPCR Master Mix (Sangon Bio, Shanghai, China), 10 μM of each primer, 10 μM of the probe, 5 ng of the DNA template, and ddH_2_O. Amplification and detection were performed using a fully automatic fluorescence quantification instrument CFX96 Touch, with an initial denaturation step at 94 °C for 3 min, followed by 40 cycles of denaturation at 94 °C for 5 s and annealing at 57 °C for 15 s.

### 2.6. Combinations Selection for Multiplex qPCR

In accordance with the fluorescence detection system of the CFX96™ Touch qPCR instrument, the probes targeting the eight distinct sites were labeled with four fluorophores—Texas Red, FAM, HEM, and CY5—and were numbered as indicated in Table 2. According to the different luminescent groups (the same fluorescent group cannot appear in the same tube), the probes of 8 milk species were combined to obtain a total of 16 combinations: 1234, 1247, 1238, 1278, 1346, 1368, 1467, 1678, 2345, 2358, 2457, 2578, 3456, 3568, 4567, 5678. To select the best combination, multiplex qPCR assays were performed. The primer and probe concentrations were maintained at 10 micromolar (μM). The upstream and downstream primer mixtures were prepared by combining each set of primers in equimolar ratios, specifically in the proportions of 1:1, 1:1:1, and 1:1:1:1, respectively. Each combination comprised four distinct types of probes, which were integrated into a homogeneous probe mixture by blending them in equal proportions, adhering to a ratio of 1:1:1:1. The DNA samples were adjusted to a concentration of 5 ng/μL, and equivalent quantities of DNA solutions from four distinct animal species were combined to serve as the amplification templates. The multiplex qPCR was performed in 20 μL reaction volumes containing 10 μL of 2× TaqMan Fast qPCR Master Mix (Sangon Bio, Shanghai, China), 10 μM of each primer, 10 μM of the probe, 5 ng of the DNA template, and ddH_2_O. Amplification was performed under the following conditions: an initial denaturation step at 94 °C for 3 min, followed by 40 cycles of denaturation at 94 °C for 5 s and annealing at 57 °C for 15 s, and extension at 72 °C for 30 s, for a total of 40 cycles, and fluorescence signals were collected during the annealing and extension phases of each cycle. All tests were performed in triplicate. Two optimal combinations were obtained by primer/probe set testing, namely system 1 including cows, buffaloes, donkeys, and camels, and system 2 including yaks, goats, sheep, and camels.

### 2.7. Multiplex PCR Specificity Test

To evaluate the specificity of the two multiplex TaqMan systems, template DNA (5 ng) from cow, buffalo, yak, goat, sheep, horse, donkey, and camel milk, and plant material were tested, along with ddH_2_O controls. Each sample was tested in triplicate.

### 2.8. The Limit of Detection (LOD) and Standard Curve of Multiplex qPCR

To determine the LOD of the developed multiplex qPCR assay, total DNA extracted from the target species was subjected to 5-fold serial dilutions. First, genomic template DNA was extracted from four target species at a concentration of 20 ng/μL with an equal mixing ratio (1:1:1:1). Subsequently, total DNA of the four species was serially diluted to concentrations of 4, 0.8, 0.16, 0.032, 0.0064, and 0.00128 ng/μL using nuclease-free water. Then, 4 μL of each diluted DNA solution was introduced into a 20 μL multi-reaction mixture so that the DNA content of each target species in the reaction mixture was maintained at 4, 0.8, 0.16, 0.032, 0.0064, and 0.00128 ng, respectively. Multiplex qPCR reactions were performed for each dilution using the selected combinations, and each template dilution was assayed in triplicate. A standard curve was constructed to evaluate the qPCR efficiency, and quantify PCR targets, extracted from the raw milk mixture with the four target species in equal proportions (1:1:1:1). Diluted samples were 5-fold serially diluted (20, 4, 0.8, 0.16, 0.032, 0.0064, 0.00128 ng/μL) with deionized distilled water and 1 µL of each added to 20 µL of reaction mixtures. The standard curve was drawn with the logarithm of concentration as the x-axis and the Ct value as the y-axis. The amplification efficiency was then determined using the formula *Eff*% = (10^−1/slope^ − 1) × 100%. PCR efficiency is deemed acceptable when it falls within the range of 90% to 110%, and the regression slope should ideally range between −3.1 and −3.6, with a coefficient of determination R^2^ of at least 0.98 [28]. According to the method proposed by Rojas et al. [29], the quantity of DNA from eight different types of target milk in unknown samples was determined based on respective Ct values, employing the equation Ct = mlog [] + C, where ‘m’ is the slope and ‘C’ is the intercept.

### 2.9. Sensitivity Test for Two Combination Systems

In order to analyze the detection limit of the established multiplex qPCR system for mixed samples, the four target milks corresponding to the preferred combination (tube 1: cow, buffalo, donkey, camel; tube 2: yak, goat, sheep, horse) were mixed in equal mass. The mixed milk was prepared with varying proportions using the non-target milk as the matrix, where the content of each target milk was set at 90%, 50%, 10%, 5%, 1%, 0.5%, 0.1%, and 0.01%, respectively. For instance, the base composition of the blend consisted of goat milk, augmented by the addition of cow milk, buffalo milk, donkey milk, and camel milk, resulting in a multifaceted composite milk amalgam. Similarly, camel milk was used as the matrix for creating another mixed milk by adding yak milk, goat milk, sheep milk, and horse milk. Using the respective matrix DNA as the negative control and ddH_2_O as the blank control, the target species components were analyzed via multiplex qPCR. Each diluted template was tested in triplicate for accuracy.

### 2.10. Repeatability Test

DNA extracted from the same batch and different batches of two combinations was prepared at several concentrations (4 ng/μL, 0.8 ng/μL, 0.16 ng/μL, 0.032 ng/μL) and used as templates. Both the intra-batch and inter-batch repeatability experiments were carried out under the optimized conditions. DNA extracts underwent serial dilution and were analyzed in duplicate. The mean cycle threshold (Ct) value, standard deviation (SD), and coefficient of variation (CV) were determined for both intra-assay and inter-assay replicates.

### 2.11. Analyses of Commercial Dairy Products

The milk components in 54 samples purchased from the commercial market were evaluated using the developed multiplex qPCR method. This study aimed to confirm the validity and reliability of the established testing protocol. A collection of 54 commercial dairy products were assembled, inclusive of 7 samples from buffalo, 15 from yak, 8 from goat, 4 from sheep, 6 from horse, 6 from donkey, and 8 from camel dairy sources. The samples encompassed a variety of types, including liquid fresh milk, yogurt, milk powder, and infant formula. The processing methodologies employed for these products ranged from pasteurization, fermentation, high-temperature sterilization, and ultra-high-temperature sterilization to high-temperature spray drying.

## 3. Results and Discussion

### 3.1. DNA Quality

DNA extraction was performed on raw milk samples, adulterated raw milk samples, and various market samples. Quantification and quality assessment of the extracted DNA were determined by measuring the absorbance at 260 nm and calculating the A260/A280 absorbance ratio. The DNA concentrations in raw milk samples and adulterated raw milk samples ranged from 10 to 100 ng/μL, whereas those in dairy product samples ranged from 5 to 50 ng/μL. The lower concentration of DNA extracted from dairy products may be due to the heat treatment during processing, which causes the DNA to fragment or degrade into smaller fragments of only a few hundred bases. DNA amplification was successfully performed in this study using multiplex qPCR. Meanwhile, the A260/A280 absorbance ratios of all extracted DNA samples were in the range of 1.8 to 2, indicating the successful extraction of high-quality DNA in all sample types [28].

### 3.2. Specificity of Primers and Probes

A rapid and efficient multiplex qPCR method was established for the simultaneous detection of cow, buffalo, yak, goat, sheep, horse, donkey, and camel in raw milk and processed products. The *Cytb* gene fragment was selected as the target gene for qPCR because of its multiple copy numbers, rapid evolution rate, intra-specific and inter-specific polymorphism, and additional protective effect on the mitochondrial membrane. Utilizing *Cytb* gene fragments can more easily help achieve lower detection limits and improve the sensitivity of qPCR detection [30]. Specificity for detecting milk-derived species increases significantly, especially in multiplex systems [31]. In addition, the specific primer sets of the target species were designed to generate shorter amplicons (104 bp–134 bp), which could selectively enhance the amplification efficiency of qPCR when identifying source components in processed dairy products.

A single qPCR detection platform was used to confirm the specificity of the primers and probes customized for the target species. Positive plasmids were used as positive controls, DNA from non-target species (soybean, corn, wheat, sweet potato, and rice) was used as negative controls, and ddH_2_O was used as a blank control. Under the optimized conditions of an annealing temperature of 57 °C, primer concentration of 10 μmol/L, and probe concentration of 10 μmol/L, amplification curves were detected only for the *Cytb* gene of cow, buffalo, yak, goat, sheep, horse, donkey, and camel, and the corresponding Ct values ranged from 20.57 to 24.93 (Table 3). There was no cross-reaction with non-target milk species, and no amplification was detected in the negative control or the blank control. The results showed that the designed primers and probes had good specificity.

### 3.3. Combinations Selection for the Multiplex qPCR System

**Figure 1:**
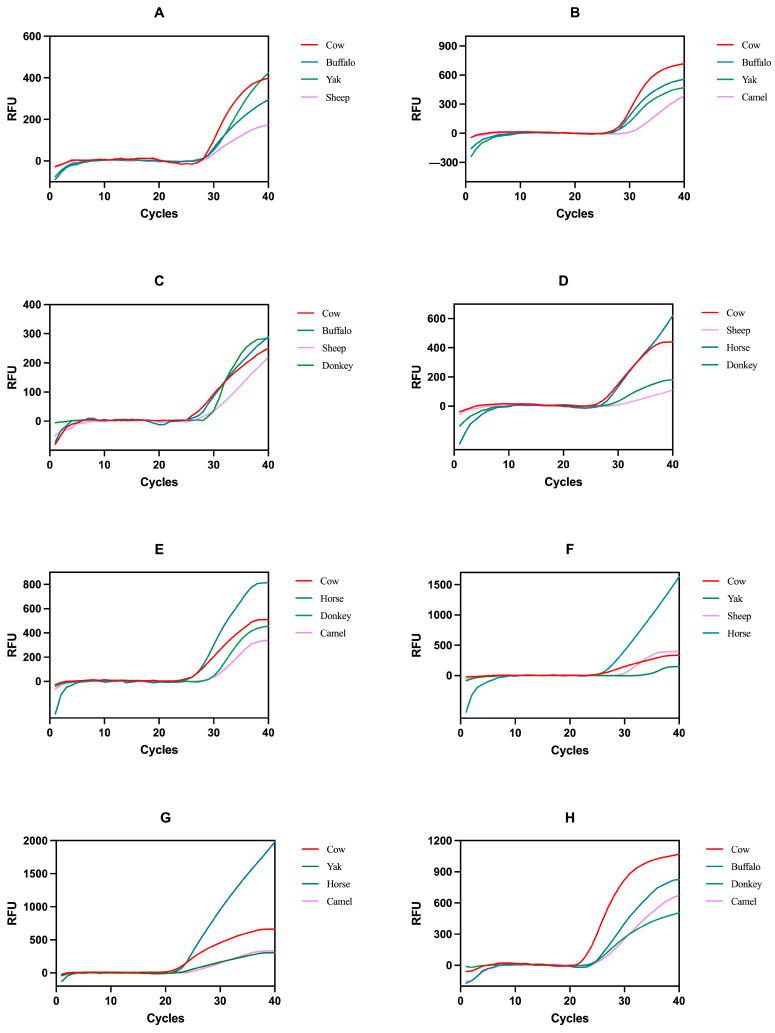

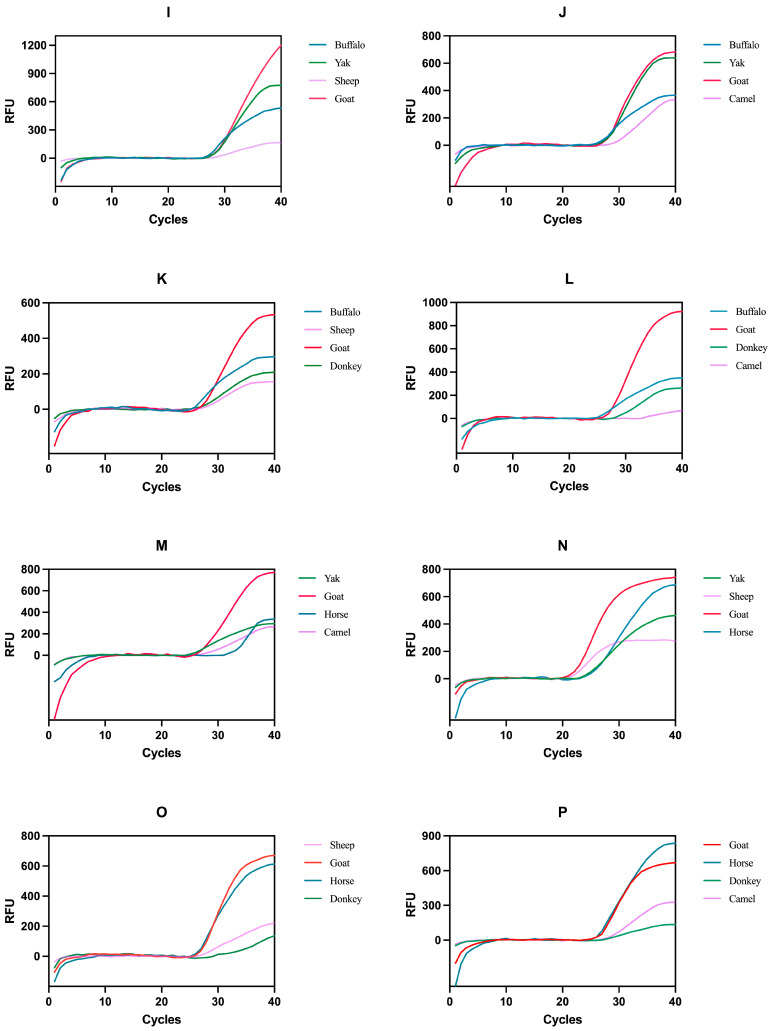
Results of multiplex qPCR amplification of 16 combinations. (**A**–**P**) 16 combinations: 1234, 1247, 1238, 1278, 1346, 1368, 1467, 1678, 2345, 2358, 2457, 2578, 3456, 3568, 4567, 5678.

Multiplex qPCR can detect the template DNA of multiple species in one reaction tube, shortening the time, reducing costs, and simplifying the experimental steps. However, multiple targets need to be specifically amplified in the same reaction system, and primer pairing and competitive amplification will affect the overall amplification effect. Therefore, multiplex qPCR cross-reaction detection was performed on 16 combinations of 8 target milk source probes according to the different luminescent groups. The results showed that all 16 combinations could detect the corresponding target species (Figure 1), but Figure 1A (yak), Figure 1C (cow, buffalo, and sheep), Figure 1D (horse, sheep), Figure 1F,G (horse), Figure 1I (goat, sheep), Figure 1K (goat), Figure 1L (camel), Figure 1O,P (donkey) all showed atypical S-shaped curves and lower RFU values, indicating that these 10 combinations had cross-reactions and amplification was inhibited. However, among the remaining reaction systems, only the combination of Figure 1H (1278) and Figure 1N (3456) could simultaneously ensure good amplification effects for the eight milk sources, indicating that there was no cross-reaction between the mixed systems. Therefore, the combination of 1278 and 3456 was selected as the optimal multiplex qPCR detection system for the eight milk sources.

**Figure 2:**
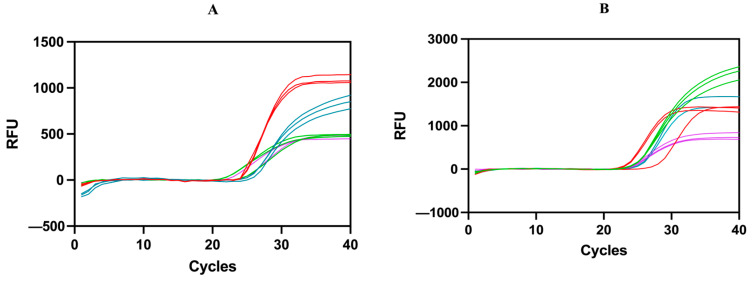
Specificity verification of multiplex qPCR. The four targets in (**A**) are red (cow), blue (buffalo), green (donkey), and purple (camel). The four targets in (**B**) are red (goat), blue (horse), green (yak), and purple (sheep). Negative and blank controls showed no amplification.

Four different fluorescent reporter dyes (FAM, HEX, Texas Red, and CY5) were used to distinguish multiple amplification products within the same reaction tube (Table 2). The single-plex qPCR systems for individual species were methodically optimized separately, employing primers and probes specific to each target species (Figure 2). Subsequently, the primers and probes for the remaining species were incrementally incorporated into the reaction mixture in a sequential manner to refine and optimize the ultimate multiplex qPCR system. The results showed that the Ct values of each species obtained using multiplex qPCR were as follows (Figure 2): cow (Ct = 20.47 ± 0.50), buffalo (Ct = 25.07 ± 0.68), yak (Ct = 22.36 ± 0.16), goat (Ct = 20.13 ± 0.06), sheep (Ct = 20.41 ± 0.81) and horse (Ct = 23.46 ± 0.26), donkey (Ct = 24.21 ± 0.81), and camel (Ct = 24.63 ± 0.93), respectively, aligned with those obtained from single qPCR systems: cow (Ct = 20.63 ± 0.26), buffalo (Ct = 25.35 ± 0.07), yak (Ct = 22.06 ± 0.04), goat (Ct = 20.53 ± 0.18), sheep (Ct = 20.43 ± 0.13), horse (Ct = 23.80 ± 0.10), donkey (Ct = 24.64 ± 0.13), and camel (Ct = 24.33 ± 0.28). The results showed that the single PCR system and the multiplex qPCR system were consistent and mutually verified.

### 3.4. Specificity of the Multiplex qPCR System

The specificity of the screened multiplex qPCR systems was evaluated in triplicate using DNA extracted from tissues of eight target species (cow, buffalo, yak, goat, sheep, horse, donkey, and camel) and five non-target species (soybean, maize, wheat, sweet potato, and rice). The primers and probes used were designed based on the *Cytb* gene of the target species, and a variety of fluorescent reporter dyes, including FAM, HEX, Texas Red, and CY5, were strategically used to accurately detect and distinguish animal-derived components in the samples. The expected results were that FAM amplified only DNA from buffalo and horse, HEX amplified only DNA from yak and donkey, Texas Red amplified only DNA from cow and goat, and CY5 amplified only DNA from sheep and camel. Mismatch sequences and dissociation temperatures (Tm) were evaluated for all primers and probes. In a multiplex qPCR system, multiple sets of primers and probes can bind to multiple templates simultaneously within a defined temperature range [32]. During the evaluation period, customized primer and probe sets for cow, buffalo, yak, goat, sheep, horse, donkey, and camel showed similar Tm ranges (58 ± 1 and 66 °C). This characteristic ensured that primers and probes bound precisely to their corresponding DNA templates within the specified experimental parameters. A primer annealing Tm of 57 °C and a higher probe Tm (66 °C) ensured primer annealing occurred before probe binding, which was critical for accurate probe testing [28]. These factors facilitated the differentiation of four fluorescent reporter dyes (FAM, HEX, Texas Red, and CY5) to distinguish four different amplifications in the same reaction mixture (Table 2). The resulting amplification curves showed species-specific amplification patterns, while background fluorescence from each species was present throughout the 40-cycle PCR assay, confirming the absence of cross-amplification (Figure 2). This observation confirmed the high specificity of the primers and probes used for the multiplex assay. The amplification signals (Ct values) obtained from the multiplex qPCR analysis for cow, buffalo, yak, goat, sheep, horse, donkey, and camel were as follows: 20.47 ± 0.50, 25.07 ± 0.68, 22.36 ± 0.16, 20.13 ± 0.06, 20.41 ± 0.81, 23.46 ± 0.26, 24.21 ± 0.81, and 24.63 ± 0.93, respectively. Meanwhile, non-target species did not show measurable Ct values (Table S1).

### 3.5. LOD and Standard Curve of Multiplex qPCR

**Figure 3:**
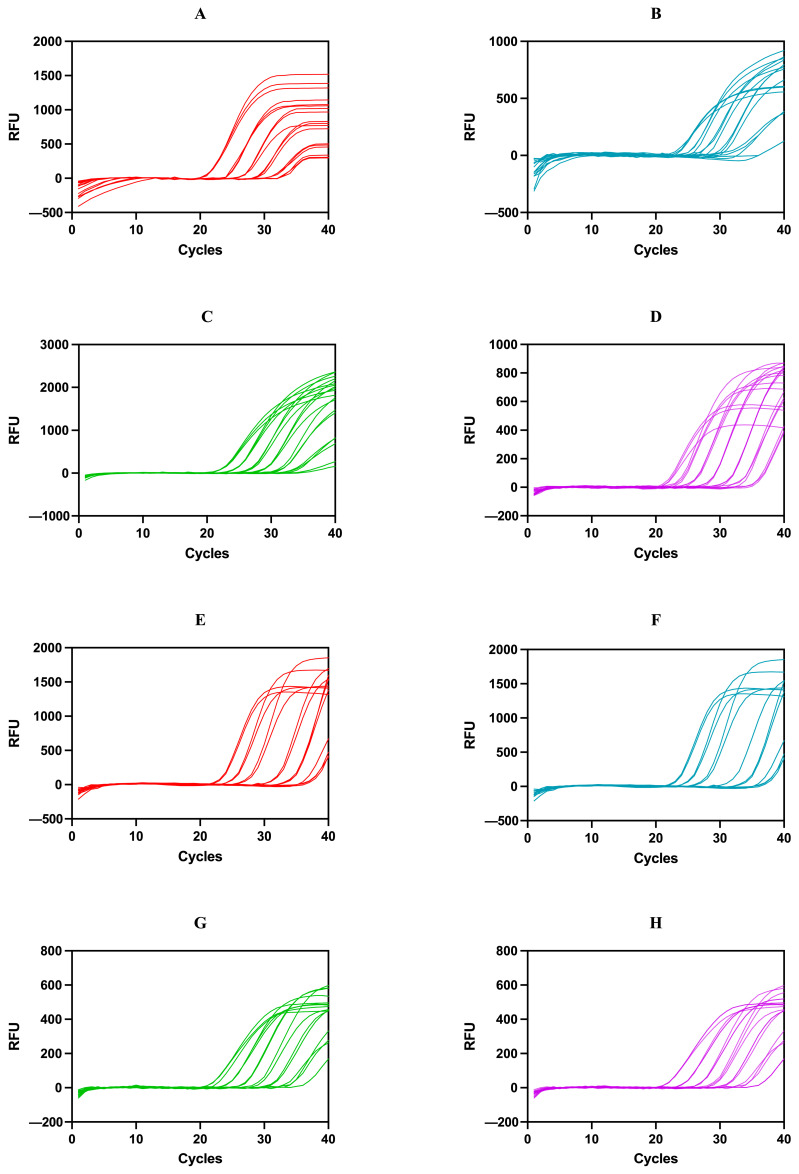
Multiplex qPCR sensitivity amplification curve of 8 milk sources. (**A**,**E**) are sensitivity amplification curves of cow and goat in Texas Red channel (red), (**B**,**F**) are sensitivity amplification curves of buffalo and horse in FAM channel (blue), (**C**,**G**) are sensitivity amplification curves of yak and donkey in HEX channel (green), and (**D**,**H**) are sensitivity amplification curves of sheep and camel in CY5 channel (purple).

LOD assists in establishing the smallest amount of the targets detectable in an adulterated specimen. To determine the LOD of the developed multiplex qPCR assay, DNA extracted from raw milk samples was serially diluted to generate a range of concentrations for use in subsequent qPCR experiments. In this study, five-fold serial dilutions (20, 4, 0.8, 0.16, 0.032, 0.0064, and 0.00128 ng/μL for each species) of mixed genomic DNA from eight target species were performed to determine the LOD of the multiplex qPCR system. The amplification curves reflected the corresponding Ct values in the system, ranging from higher to lower concentrations (Figure 3). Table 4 lists the mean Ct values and inter-day relative standard deviations (RSDs) for different raw milk products, showing the observed Ct values over a range of DNA concentrations, from 20 ng/μL to 0.00128 ng/μL. The results showed that the multiplex qPCR system was able to detect and quantify at least 0.0064 ng of DNA in cow and buffalo milk, and at least 0.00128 ng of DNA in yak, goat, sheep, horse, donkey, and camel milk. The RSDs for all diluted DNA samples were less than 2.0 (ranging from 0.03 to 1.85). Guo et al. [22] described the detection of 0.001 ng of bovine and equine DNA in milk and dairy products based on species-specific *Taq*Man probes, confirming that triplex PCR testing was a time-saving and cost-saving technology. Tichy et al. [33] developed chia- and quinoa-specific primer/probe sets based on TaqMan technology and demonstrated that chia seeds and quinoa could be detected even in trace amounts of seed material less than 0.1%. A fast duplex qPCR assay developed by Kim et al. [31] can identify chicken and pigeon DNA levels as low as 0.1 pg, suggesting that this method may be suitable for verifying the authenticity of the presence of pigeon and chicken in meat products. Agrimonti et al. [15] demonstrated that a SYBR green-based quadruple qPCR assay could detect at least 0.02 ng of milk DNA. All of these studies have shown that LODs vary between species and are affected by various factors, including the degree of digestion, processing conditions, sample age, and the composition of the background matrix. Currently, there are few systematic methods to quickly and accurately identify the authenticity of milk from different animal sources (cow, buffalo, yak, goat, sheep, horse, donkey, and camel). However, we have effectively developed a multiplex qPCR technology capable of simultaneously detecting adulterants from eight major milk sources, reducing the LOD to 0.00128 ng DNA, which significantly enhances the sensitivity of milk source identification.

### 3.6. Quantification and Efficiency of Multiplex qPCR

**Figure 4:**
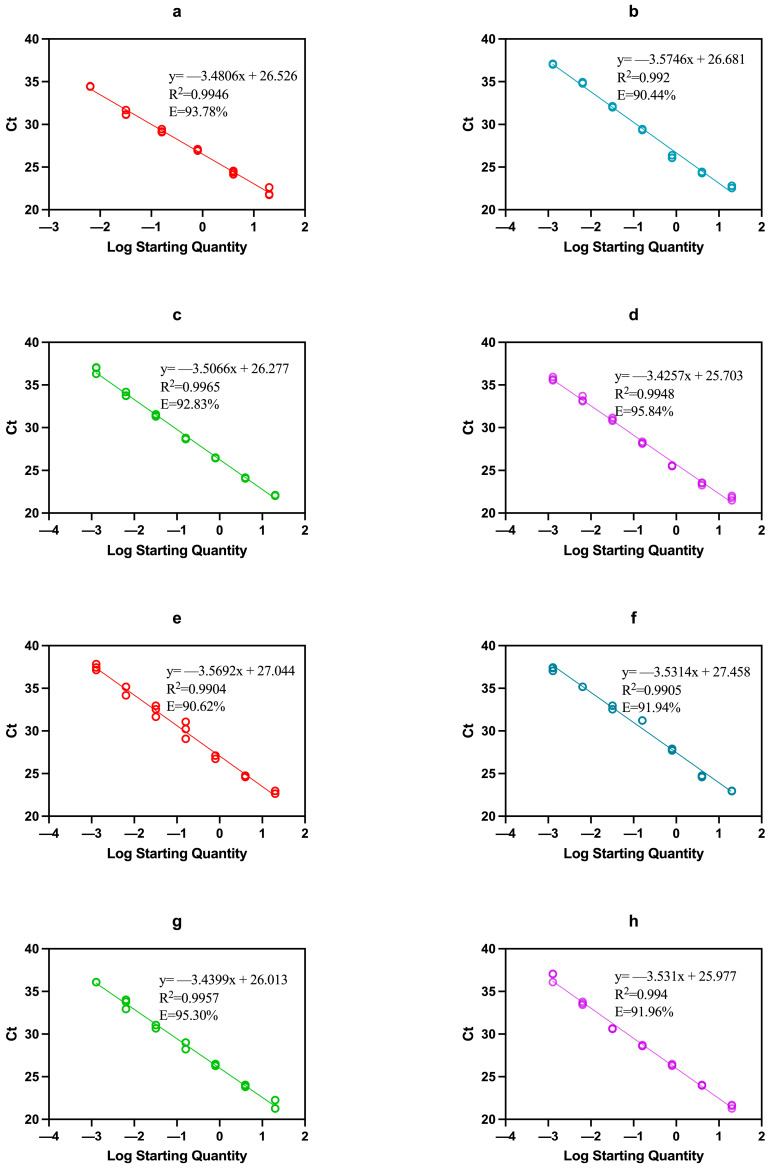
Multiplex qPCR standard curves of milk sources; (**a**–**h**) are the standard curves of dairy cow, buffalo, yak, sheep, goat, horse, donkey, and camel milk source in multiplex qPCR amplification.

To quantify the DNA of each target species, DNA (20 ng/μL) extracted from an equal amount of raw milk mixture (1:1:1:1) of each target species was serially diluted five times to obtain total DNA concentrations of 4, 0.8, 0.16, 0.032, 0.0064, and 0.00128 ng in the reaction mixture. For comparison and graphical representation, standard curves for each species were constructed using the ggplot2 package in R (V3.3.0; http://ggplot2.tidyverse.org, accessed on 10 October 2023) (Figure 4). The standard curves showed robust linear regression with regression coefficients (R^2^) of 0.9946, 0.9920, 0.9965, 0.9948, 0.9904, 0.9905, 0.9957, and 0.9940 for cow, buffalo, yak, goat, sheep, horse, donkey, and camel, respectively. The slopes associated with each standard curve were determined as follows: −3.4806, −3.5746, −3.5066, −3.4257, −3.5692, −3.5314, −3.4399, and −3.5310, respectively. The calculated PCR efficiencies reached 93.78% (cow), 90.44% (buffalo), 92.83% (yak), 95.84% (goat), 90.62% (sheep), 91.94% (horse), 95.30% (donkey), and 91.96% (camel). The regression coefficient, correlation slope, and PCR efficiency were all within the recommended parameter range proposed by Sultana et al. [28], demonstrating that this multiplex qPCR detection method has high amplification efficiency and good linearity. Therefore, the generated standard curve and multiplex qPCR system can achieve satisfactory results for the quantitative detection of the contribution of target species in mixed milk samples. The findings of Cheng et al. [32] also support the results of this study. The multiplex qPCR method they established has amplification efficiencies of 104.38%, 91.75%, and 97.46% for chicken, duck, and pig components. Iwobi et al. [34] observed multiplex qPCR efficiency of 101.1% and 91.6% for beef and pork, respectively. Similarly, Sultana et al. [28] calculated 110%, 109.78%, and 105.06% efficiencies for bovine, porcine, and fish, respectively.

### 3.7. Detection Limit of Adulteration

The sensitivity of the established multiplex qPCR system to detect the content of eight target milks in adulterated multi-ingredient milk mixture models was evaluated. Adulteration levels of eight species were detected at 0.01–5% within the multicomponent mixture (Table 5). For the eight target species, the lowest detectable Ct values of cow milk, yak milk, goat milk, sheep milk, and camel milk (0.01%) ranged from 27.036 ± 0.200 to 34.568 ± 0.068. The lowest detectable Ct value for buffalo milk and horse milk (0.5%) were 33.948 ± 0.415 and 33.547 ± 0.242, respectively. The lowest detectable Ct value for donkey milk (5%) was 35.628 ± 0.099. The RSDs were calculated according to the average Ct value of simulated adulterated dairy products with different addition levels, ranging from 0.09% to 3.62%. These findings unequivocally illustrate the robust sensitivity, specificity, and reliability of the multiplex qPCR system developed, reliably detecting adulteration from bovine species at concentrations as low as 0.01%. Compared with Genis et al. [35], who could detect a minimum of 3.3% dairy products based on synchronous fluorescence spectroscopy, our method had the advantages of high throughput, high speed, accuracy, and strong specificity. The multiplex qPCR system established by Cottenet et al. [19] could identify adulterated cow milk in buffalo milk with a LOD of 1%, suggesting that this method can effectively replace PCR-RFLP and multiplex amplification technology for species identification in mixed foods. Therefore, the multiplex qPCR method established in this experiment exhibits high sensitivity, overcoming the challenges of traditional PCR false positive and cross-contamination, while enabling automated real-time detection and analysis. Furthermore, it demonstrates stronger specificity and sensitivity compared with the fluorescent dye method, becoming a commonly used molecular biological detection method in the identification of animal-derived components [36].

### 3.8. Repeatability

The established multiplex qPCR method was used to assess the intra-assay and inter-assay repeatability across four DNA dilution concentrations (4 ng/μL, 0.8 ng/μL, 0.16 ng/μL, 0.032 ng/μL) (Table 6 and Table 7). Each dilution concentration was repeated three times for both intra-assay and inter-assay repeatability tests. Intra-batch analysis comprised three repeated operations conducted within a single day, whereas inter-batch analysis involved data collected over three consecutive days. Precision was evaluated through the determination of standard deviation (SD) and relative standard deviation (RSD). The results showed that the intra-batch RSD values of eight milk DNAs were less than 5.201%, and the inter-batch RSD values were less than 3.107%. Moreover, the intra-batch and inter-batch RSD values were less than 10%, suggesting that multiplex qPCR detection method established in this experiment had good stability and repeatability.

### 3.9. Actual Sample Detection

Critical to ensuring a healthy and sustainable dairy market is the certification of dairy ingredients. Accurate labeling for products can play an important in preventing or monitoring the proliferation of substandard dairy alternatives, thereby bolstering consumer confidence. Since the various treatments affecting the DNA degradation and the complex matrix of processed dairy products, 54 commercially processed dairy products produced by different heat treatment were selected for market sample testing (Table 8). The amplification results indicated that the Ct values of all dairy products were less than 35, which proved that the established multiplex qPCR method could meet the requirements of genes from dairy products with different processing methods. This developed multiplex qPCR method was used to verify the authenticity of eight animal milk sources. The findings revealed that 18 dairy products detected unidentified species, with an adulteration rate of 33.33%. Primarily, this adulteration was observed in buffalo, yak, goat, sheep, and donkey milk products. Specifically, four out of seven buffalo dairy products were found to contain cow ingredients without being labeled, indicating a discrepancy rate of 57.14%. Moreover, in 8 out of 15 yak dairy products, the presence of cow ingredients or goat ingredients were found, resulting in a discrepancy rate of 53.33%. Furthermore, two out of eight goat dairy products, one out of four sheep dairy products, and three out of six donkey dairy products were found to contain cow ingredients, with discrepancy rates of 25%, 25%, and 50%, respectively. Notably, horse and camel dairy products did not exhibit any discrepancies, which complied with their milk source labels.

## 4. Conclusions

This method used multi-copy mitochondrial genes as target genes, referring to the highly conserved intra-specific intermediate region of *Cytb* in the mitochondrial genome of the target milk-derived species as the target sequence for specific detection of each milk-derived species. By introducing specific fluorescent probes into the reaction system to identify and detect the amplified products, and optimizing the concentrations of primers and probes, annealing temperature, and other conditions, the multiplex qPCR system for the simultaneous detection of milk components of cows, buffaloes, yaks, goats, sheep, horses, donkeys, and camels was established. The system could amplify eight target sources in two reaction tubes concurrently, effectively shortening and simplifying the process of adulteration identification of dairy products, thereby improving the detection efficiency. In addition, this method demonstrated robust specificity and sensitivity, with no cross-reactivity for 10 non-target sources, which could detect concentrations ranging from 0.00128 to 0.0064 ng/μL. Since the detection limit of 0.01~5% of milk content for adulteration in raw milk, the detection results are accurate and stable. Additionally, it is applicable for detecting animal-derived constituents in high-quality dairy products, such as yak milk and camel milk. Considering that economically motivated adulteration usually exceeds 10%, the developed methodology can serve as a valuable tool for discerning potential adulteration in characteristic milk samples. In summary, the multiplex qPCR system developed in this study represents an efficacious detection approach for ascertaining the authenticity of milk and dairy products available in the market, thereby furnishing technical reinforcement in combating the adulteration and falsification of illicit dairy products. This initiative aids in enhancing market oversight mechanisms and upholding food safety standards for consumers.

## Figures and Tables

**Table 1:** Primer and probe sequence.

Number	Target Species	Sequence (5′-3′)	Amplicon/bp
1	Cow	F: CCTAGCAATACACTACACATCCG R: TTGAAGCTCCGTTTGCGT cow-P: Texas Red-TCTGTTACCCATATCTGCCGAGACGTG-BHQ2 buffalo-P: FAM-CGTGAACTATGGATGAA-MGB yak-P: HEX-CTCCGTTGCCCATAT-MGB	106
2	Buffalo		
3	Yak		
4	Sheep	F: ATAGGCTATGTTTTACCATGAGGAC R: CATTCGACTAGGTTTGTGCCA sheep-P:CY5-TATTACCAACCTCCTTTC-MGB goat-P: Texas Red-ACAGTCATCACTAATCTTCTTTCAGCAATCCC-BHQ2	104
5	Goat		
6	Horse	F: AGACCCAGACAACTACACCCC R: TTGTTGGGAATGGAGCGTA horse-P: FAM-TACTTCCTGTTTGCCTAC-MGB donkey-P: HEX-TTCCTATTTGCTTACGCC-MGB	108
7	Donkey		
8	Camel	F: ACAGGCTCTAATAACCCGACAG R: GGTGAGAACAGTACGAGAATAAGG camel-P:CY5- CTCCTCAGACATAGACA-MGB	134

Notes: F stands for upstream primers, R for downstream primers, and P for probe.

**Table 2:** Combinations for the multiplex qPCR assays.

Probe Dye	Multiplex 1	Multiplex 2
Texas Red	Cow	Goat
FAM	Buffalo	Horse
HEX	Donkey	Yak
CY5	Camel	Sheep

**Table 3:** Single qPCR detection of specific primers and probes.

Sample	Ct Value								
	Positive Control	Cow	Buffalo	Yak	Goat	Sheep	Horse	Donkey	Camel
Cow	20.57 ± 0.21	20.63 ± 0.26	-	-	-	-	-	-	-
Buffalo	25.34 ± 0.46	-	25.35 ± 0.07	-	-	-	-	-	-
Yak	22.05 ± 0.02	-	-	22.06 ± 0.04	-	-	-	-	-
Goat	20.99 ± 0.37	-	-	-	20.53 ± 0.18	-	-	-	-
Sheep	20.73 ± 0.22	-	-	-	-	20.43 ± 0.13	-	-	-
Horse	23.20 ± 1.31	-	-	-	-	-	23.80 ± 0.10	-	-
Donkey	24.93 ± 0.24	-	-	-	-	-	-	24.64 ± 0.13	-
Camel	24.27 ± 0.00	-	-	-	-	-	-	-	24.33 ± 0.28
Negative control	-	-	-	-	-	-	-	-	-
Blank control	-	-	-	-	-	-	-	-	-

Notes: “-” is no increase of the fluorescence signal within 40 cycles.

**Table 4:** Ct values of each target species obtained from the amplification plot with a 5-fold serially diluted DNA of each target species for the determination of LOD.

DNA Concentration (ng)	Cow				Buffalo				Donkey				Camel			
	Ct Value	Mean Ct Value	SD	RSD (%)	Ct Value	Mean Ct Value	SD	RSD (%)	Ct Value	Mean Ct Value	SD	RSD (%)	Ct Value	Mean Ct Value	SD	RSD (%)
20	21.741	21.961	0.262	1.19	22.807	22.755	0.070	0.31	21.268	21.760	0.402	1.85	21.597	21.517	0.178	0.83
	21.813				22.801				22.253				21.683			
	22.330				22.656				21.760				21.270			
4	24.148	24.345	0.149	0.61	24.441	24.365	0.062	0.25	24.007	23.953	0.103	0.43	24.007	24.045	0.031	0.13
	24.375				24.363				24.044				24.044			
	24.510				24.290				23.808				24.084			
0.8	27.015	27.045	0.048	0.18	26.394	26.185	0.148	0.57	26.282	26.371	0.086	0.33	26.371	26.332	0.037	0.14
	27.113				26.070				26.487				26.282			
	27.008				26.090				26.343				26.343			
0.16	29.176	29.232	0.150	0.51	29.452	29.384	0.048	0.16	28.228	28.612	0.322	1.12	28.612	28.607	0.009	0.03
	29.083				29.351				28.594				28.594			
	29.437				29.350				29.015				28.615			
0.032	31.139	31.289	0.182	0.58	32.115	32.089	0.063	0.20	30.114	30.612	0.385	1.26	30.612	30.632	0.027	0.09
	31.183				32.002				30.669				30.614			
	31.546				32.149				31.052				30.669			
0.0064	34.460	34.460	0.011	0.03	34.812	34.885	0.053	0.15	32.943	33.588	0.465	1.38	33.588	33.606	0.151	0.45
	34.474				34.908				33.799				33.430			
	34.447				34.935				34.022				33.799			
0.00128	-	-	-	-	-	-	-	-	36.536	36.526	0.356	0.98	37.026	36.732	0.455	1.24
	-				-				36.090				37.080			
	-				-				36.962				36.090			
**DNA Concentration (ng)**	**Yak**				**Goat**				**Sheep**				**Horse**			
	**Ct Value**	**Mean Ct Value**	**SD**	**RSD (%)**	**Ct Value**	**Mean Ct Value**	**SD**	**RSD (%)**	**Ct Value**	**Mean Ct Value**	**SD**	**RSD (%)**	**Ct Value**	**Mean Ct Value**	**SD**	**RSD (%)**
20	22.064	22.076	0.025	0.11	22.970	22.747	0.157	0.69	21.805	21.733	0.180	0.83	22.935	22.947	0.016	0.07
	22.111				22.635				21.486				22.970			
	22.054				22.635				21.908				22.935			
4	24.147	24.219	0.058	0.24	24.765	24.802	0.052	0.21	23.577	23.446	0.141	0.60	24.587	24.646	0.084	0.34
	24.290				24.765				23.510				24.587			
	24.219				24.876				23.250				24.765			
0.8	26.476	26.419	0.123	0.47	27.109	26.983	0.177	0.66	25.592	25.534	0.041	0.16	27.709	27.783	0.089	0.32
	26.504				26.733				25.502				27.733			
	26.278				27.109				25.507				27.909			
0.16	28.692	28.621	0.090	0.31	30.069	29.640	0.421	1.42	28.209	28.155	0.041	0.14	31.220	31.074	0.206	0.66
	28.677				29.069				28.145				31.220			
	28.494				29.783				28.111				30.783			
0.032	31.308	31.226	0.138	0.44	32.965	32.787	0.181	0.55	30.933	30.860	0.057	0.19	32.539	32.787	0.181	0.55
	31.338				32.539				30.793				32.965			
	31.031				32.856				30.854				32.856			
0.0064	33.725	33.611	0.191	0.57	35.175	34.842	0.471	1.35	33.197	33.334	0.270	0.81	35.475	35.342	0.125	0.35
	33.766				34.175				33.711				35.175			
	33.342				35.175				33.095				35.375			
0.00128	37.011	37.040	0.021	0.06	37.444	37.206	0.173	0.46	35.665	35.573	0.068	0.19	37.444	37.286	0.177	0.48
	37.051				37.138				35.548				37.375			
	37.060				37.038				35.505				37.038			

Notes: SD—standard deviation; RSD—relative standard deviation.

**Table 5:** Mean Ct values and inter-day RSD of different model milk products.

Products	Spike Level (%)	Mean Ct Value			SD	RSD
		Day 1	Day 2	Day 3		
Cow	90	20.130	19.200	19.270	0.518	2.651
	50	20.110	20.210	20.210	0.058	0.286
	10	23.880	23.300	23.780	0.310	1.311
	5	24.070	24.040	24.380	0.188	0.779
	1	27.240	27.820	27.900	0.360	1.303
	0.5	28.560	28.430	29.630	0.659	2.281
	0.1	29.160	29.750	29.610	0.308	1.045
	0.01	32.180	32.690	31.550	0.571	1.777
Buffalo	90	20.321	20.243	20.399	0.078	0.38
	50	21.640	22.020	21.182	0.420	1.94
	10	22.321	22.785	21.228	0.799	3.62
	5	21.621	22.699	22.042	0.543	2.46
	1	24.597	24.656	24.626	0.030	0.12
	0.5	33.948	34.363	33.534	0.415	1.22
	0.1	-	-	-	-	-
	0.01	-	-	-	-	-
Donkey	90	22.647	22.305	22.990	0.343	1.51
	50	23.158	22.691	23.492	0.402	1.74
	10	31.946	31.975	31.917	0.029	0.09
	5	35.530	35.628	35.727	0.099	0.28
	1	-	-	-	-	-
	0.5	-	-	-	-	-
	0.1	-	-	-	-	-
	0.01	-	-	-	-	-
Camel	90	24.690	23.980	23.780	0.478	1.980
	50	23.182	23.182	23.414	0.134	0.576
	10	23.411	23.865	23.700	0.230	0.972
	5	24.691	25.041	24.340	0.350	1.419
	1	25.492	26.433	25.749	0.487	1.879
	0.5	27.090	27.029	26.400	0.382	1.423
	0.1	32.862	31.507	32.469	0.697	2.160
	0.01	34.568	34.499	34.636	0.068	0.198
Yak	90	22.243	21.545	21.894	0.349	1.596
	50	22.755	22.849	22.427	0.221	0.976
	10	24.289	24.134	24.218	0.078	0.321
	5	26.187	26.990	26.463	0.408	1.536
	1	27.150	26.674	26.536	0.322	1.202
	0.5	27.439	27.370	27.765	0.211	0.766
	0.1	28.253	28.043	28.083	0.112	0.397
	0.01	29.619	29.945	29.058	0.449	1.520
Goat	90	18.746	18.434	18.407	0.189	1.018
	50	19.977	19.962	19.839	0.076	0.381
	10	22.611	23.038	22.706	0.458	1.981
	5	23.665	23.764	23.841	0.590	2.555
	1	25.814	24.607	24.576	0.706	2.822
	0.5	26.661	26.016	25.801	0.488	1.711
	0.1	27.099	26.363	26.207	0.476	1.793
	0.01	29.826	29.508	29.865	0.196	0.659
Sheep	90	19.493	19.694	19.413	0.145	0.741
	50	21.906	21.277	21.159	0.401	1.872
	10	21.634	21.758	20.557	0.661	3.099
	5	24.486	23.445	23.238	0.669	2.821
	1	26.217	26.103	26.228	0.069	0.264
	0.5	24.486	24.330	24.521	0.102	0.416
	0.1	27.289	27.335	27.310	0.023	0.085
	0.01	26.834	27.234	27.040	0.200	0.740
Horse	90	22.443	22.424	22.521	0.052	0.230
	50	23.258	23.132	23.279	0.080	0.343
	10	25.202	25.816	25.365	0.318	1.249
	5	26.116	25.607	26.126	0.297	1.144
	1	27.776	28.281	27.865	0.269	0.963
	0.5	33.321	33.803	33.517	0.242	0.722
	0.1	-	-	-	-	-
	0.01	-	-	-	-	-

Notes: SD—standard deviation; RSD—relative standard deviation; “-” is no increase of the fluorescence signal within 40 cycles.

**Table 6:** Multiplex qPCR intra-batch repeatability test.

DNA Concentration/ng/μL	Cow		Buffalo		Donkey		Camel	
	Mean ± SD	*RSD*/%	Mean ± SD	*RSD*/%	Mean ± SD	*RSD*/%	Mean ± SD	*RSD*/%
4	24.149 ± 0.514	2.129	24.441 ± 0.821	3.357	25.461 ± 0.404	1.586	23.953 ± 0.127	0.528
0.8	24.441 ± 0.821	3.357	26.394 ± 1.373	5.201	28.519 ± 0.144	0.506	26.371 ± 0.105	0.399
0.16	29.466 ± 0.305	1.034	29.452 ± 0.653	2.217	31.762 ± 0.299	0.941	28.612 ± 0.394	1.377
0.032	32.139 ± 0.572	1.780	32.449 ± 0.830	2.559	33.337 ± 0.996	2.989	30.612 ± 0.472	1.541
**DNA Concentration/ng/μL**	**Yak**		**Goat**		**Sheep**		**Horse**	
	**Mean ± SD**	***RSD*/%**	**Mean ± SD**	***RSD*/%**	**Mean ± SD**	***RSD*/%**	**Mean ± SD**	***RSD*/%**
4	24.147 ± 0.078	0.325	26.809 ± 0.269	1.005	23.557 ± 0.364	1.545	24.507 ± 0.176	0.718
0.8	26.476 ± 0.214	0.810	31.024 ± 0.222	0.714	25.592 ± 0.151	0.592	28.079 ± 0.066	0.236
0.16	28.692 ± 0.173	0.601	33.787 ± 0.221	0.655	28.209 ± 0.141	0.498	32.623 ± 0.069	0.212
0.032	31.308 ± 0.263	0.841	35.238 ± 0.063	0.178	30.933 ± 0.192	0.620	36.434 ± 0.399	1.094

Notes: SD—standard deviation; RSD—relative standard deviation.

**Table 7:** Multiplex qPCR inter-batch repeatability test.

DNA Concentration/ng/μL	Cow		Buffalo		Donkey		Camel	
	Mean ± SD	*RSD*/%	Mean ± SD	*RSD*/%	Mean ± SD	*RSD*/%	Mean ± SD	*RSD*/%
4	23.899 ± 0.724	3.031	24.338 ± 0.252	1.035	26.201 ± 0.346	1.322	23.335 ± 0.287	1.230
0.8	27.011 ± 0.116	0.430	27.036 ± 0.296	1.095	28.516 ± 0.587	2.058	26.493 ± 0.505	1.905
0.16	29.411 ± 0.328	1.115	29.864 ± 0.588	1.967	31.881 ± 0.526	1.651	28.762 ± 0.615	2.137
0.032	31.487 ± 0.237	0.752	32.038 ± 0.105	0.327	34.073 ± 0.188	0.553	31.143 ± 0.127	0.407
**DNA Concentration/ng/μL**	**Yak**		**Goat**		**Sheep**		**Horse**	
	**Mean ± SD**	***RSD*/%**	**Mean ± SD**	***RSD*/%**	**Mean ± SD**	***RSD*/%**	**Mean ± SD**	***RSD*/%**
4	24.142 ± 0.153	0.634	26.459 ± 0.179	0.677	23.535 ± 0.332	1.410	24.247 ± 0.185	0.761
0.8	26.489 ± 0.526	1.986	29.956 ± 0.259	0.863	25.309 ± 0.440	1.739	28.254 ± 0.306	1.082
0.16	28.767 ± 0.450	1.564	33.276 ± 0.005	0.014	28.438 ± 0.180	0.633	32.433 ± 1.008	3.107
0.032	31.577 ± 0.445	1.410	35.742 ± 0.074	0.208	31.299 ± 0.512	1.637	36.423 ± 0.219	0.601

Notes: SD—standard deviation; RSD—relative standard deviation.

**Table 8:** Detection of species composition of dairy animals from commercial dairy products.

Number	Milk Source	Milk Source Identification	Detection of Milk Source	Ct Value		
				Milk Source	Cow	Goat
1	Buffalo	Raw buffalo milk	buffalo		22.68	
2		Pure buffalo milk powder	cow	23.10		
3		Pure buffalo milk	cow	20.74		
4		Pure buffalo milk	buffalo		24.09	
5		Pure buffalo milk	cow	25.2		
6		Pure buffalo milk	cow	18.3		
7		Pure buffalo milk	buffalo		18.9	
8	Yak	Full-fat yak milk	cow, goat		18.33	27.00
9		Full-fat yak milk	cow, goat		20.61	26.67
10		Full-fat yak milk	yak	22.86		
11		Full-fat yak milk	yak	19.81		
12		Full-fat yak milk	cow, goat		20.50	28.34
13		Pure milk	cow, goat		22.63	28.32
14		Organic pure milk	yak	23.52		
15		Full-fat yak milk powder	cow, goat		18.32	30.80
16		Pure yak milk powder	yak	23.38		
17		Pure yak milk powder	yak, cow, goat	23.26	20.07	28.97
18		Pure yak milk powder	yak, cow, goat	23.38	19.60	28.11
19		Pure yak milk powder	yak, cow, goat	22.72	20.05	28.27
20		Pure milk	yak	25.66		
21		Pure milk	yak	18.70		
22		Pure milk	yak	24.88		
23	Goat	Goat milk powder	goat, cow	25.52	25.08	
24		Full-fat goat milk	goat, cow	25.66	19.99	
25		Pure goat powder	goat	20.68		
26		Pasteurized goat milk	goat	18.99		
27		Pasteurized goat milk	goat	17.30		
28		Pasteurized goat milk	goat	21.81		
29		Pure goat powder	goat	18.71		
30		Pure goat powder	goat	18.87		
31	Sheep	Full-fat goat milk powder	sheep	22.18		
32		Full-fat goat milk powder	sheep	23.29		
33		Pasteurized sheep milk	sheep	23.43		
34		Pasteurized sheep milk	sheep, cow	25.43	21.86	
35	Horse	Horse milk wine	horse	31.53		
36		Horse milk wine	horse	24.14		
37		Sour horse milk	horse	24.77		
38		Sour horse milk	horse	24.52		
39		Sour horse milk	horse	25.72		
40		Sour horse milk	horse	26.02		
41	Donkey	Fresh donkey milk	cow		19.96	
42		Whole milk powder	cow		19.76	
43		Whole milk powder	donkey	24.83		
44		Whole milk powder	cow		19.43	
45		Fresh donkey milk	donkey	28.32		
46		Whole milk powder	donkey	25.18		
47	Camel	Pure camel milk	camel	25.04		
48		Fresh camel milk	camel	22.69		
49		Pure camel milk	camel	20.42		
50		Whole milk powder	camel	23.01		
51		Whole milk powder	camel	21.24		
52		Sterilized camel milk	camel	21.91		
53		Pure camel powder	camel	23.99		
54		Pure camel powder	camel	22.22		

## Data Availability

The original contributions presented in the study are included in the article/Supplementary Material, further inquiries can be directed to the corresponding author.

## Supplementary Materials

The following supporting information can be downloaded at: https://www.mdpi.com/article/10.3390/foods13203213/s1, Table S1: Specificity test of multiplex qPCR assay.

## References

[1] Amalfitano N., Patel N., Haddi M.-L., Benabid H., Pazzola M., Vacca G.M., Tagliapietra F., Schiavon S., Bittante G. (2024). Detailed mineral profile of milk, whey, and cheese from cows, buffaloes, goats, ewes and dromedary camels, and efficiency of recovery of minerals in their cheese. J. Dairy Sci..

[2] Cimmino F., Catapano A., Villano I., Di Maio G., Petrella L., Traina G., Pizzella A., Tudisco R., Cavaliere G. (2023). Invited review: Human, cow, and donkey milk comparison: Focus on metabolic effects. J. Dairy Sci..

[3] Nayik G.A., Jagdale Y.D., Gaikwad S.A., Devkatte A.N., Dar A.H., Dezmirean D.S., Bobis O., Ranjha M.M.A.N., Ansari M.J., Hemeg H.A. (2021). Recent insights into processing approaches and potential health benefits of goat milk and its products: A review. Front. Nutr..

[4] Sepe L., Argüello A. (2019). Recent advances in dairy goat products. Asian-Australas. J. Anim. Sci..

[5] Di Domenico M., Di Giuseppe M., Rodríguez J.W., Cammà C. (2017). Validation of a fast real-time PCR method to detect fraud and mislabeling in milk and dairy products. J. Dairy Sci..

[6] Zhang J., Wei L., Miao J., Yu Y., Yu N., Hu Q., Chen H., Chen Y. (2024). Authenticity identification of animal species in characteristic milk by integration of shotgun proteomics and scheduled multiple reaction monitoring (MRM) based on tandem mass spectrometry. Food Chem..

[7] Windarsih A., Arifah M.F., Suratno, Rohman A. (2023). The application of untargeted metabolomics using UHPLC-HRMS and chemometrics for authentication of horse milk adulterated with cow milk. Food Anal. Methods.

[8] Zhou C., Liu L., Chen J., Fu Q., Chen Z., Wang J., Sun X., Ai L., Xu X., Wang J. (2024). Rapid authentication of characteristic milk powders by recombinase polymerase amplification assays. Food Chem..

[9] Bansal S., Singh A., Mangal M., Mangal A.K., Kumar S. (2017). Food adulteration: Sources, health risks, and detection methods. Crit. Rev. Food Sci. Nutr..

[10] Flynn K., Villarreal B.P., Barranco A., Belc N., Björnsdóttir B., Fusco V., Rainieri S., Smaradóttir S.E., Smeu I., Teixeira P. (2019). An introduction to current food safety needs. Trends Food Sci. Technol..

[11] Sobrino-Gregorio L., Vilanova S., Prohens J., Escriche I. (2019). Detection of honey adulteration by conventional and real-time PCR. Food Control.

[12] Shabani H., Mehdizadeh M., Mousavi S.M., Dezfouli E.A., Solgi T., Khodaverdi M., Rabiei M., Rastegar H., Alebouyeh M. (2015). Halal authenticity of gelatin using species-specific PCR. Food Chem..

[13] Abdel-Rahman S., Ahmed M. (2007). Rapid and sensitive identification of buffalo’s, cattle’s and sheep’s milk using species-specific PCR and PCR–RFLP techniques. Food Control.

[14] Yu W., Chen Y., Wang Z., Qiao L., Xie R., Zhang J., Bian S., Li H., Zhang Y., Chen A. (2021). Multiple authentications of high-value milk by centrifugal microfluidic chip-based real-time fluorescent LAMP. Food Chem..

[15] Agrimonti C., Pirondini A., Marmiroli M., Marmiroli N. (2015). A quadruplex PCR (qxPCR) assay for adulteration in dairy products. Food Chem..

[16] Li J., Cheng J., Li S., Wu J.J., Li J. (2023). Virtual Multiplexing Chamber-Based Digital PCR for Camel Milk Authentication Applications. Micromachines.

[17] Mohamad N.A., El Sheikha A.F., Mustafa S., Mokhtar N.F.K. (2013). Comparison of gene nature used in real-time PCR for porcine identification and quantification: A review. Food Res. Int..

[18] Fajardo V., González I., Martín I., Rojas M., Hernández P.E., García T., Martín R. (2008). Real-time PCR for detection and quantification of red deer (*Cervus elaphus*), fallow deer (*Dama dama*), and roe deer (*Capreolus capreolus*) in meat mixtures. Meat Sci..

[19] Cottenet G., Blancpain C., Golay P.-A. (2011). Simultaneous detection of cow and buffalo species in milk from China, India, and Pakistan using multiplex real-time PCR. J. Dairy Sci..

[20] Sentandreu M., Sentandreu E. (2014). Authenticity of meat products: Tools against fraud. Food Res. Int..

[21] Hossain M.M., Ali M.E., Sultana S., Asing Bonny S.Q., Kader M.A., Rahman M.A. (2017). Quantitative tetraplex real-time polymerase chain reaction assay with TaqMan probes discriminates cattle, buffalo, and porcine materials in food chain. J. Agric. Food Chem..

[22] Guo L., Qian J.-P., Guo Y.-S., Hai X., Liu G.-Q., Luo J.-X., Ya M. (2018). Simultaneous identification of bovine and equine DNA in milks and dairy products inferred from triplex TaqMan real-time PCR technique. J. Dairy Sci..

[23] Dooley J.J., Paine K.E., Garrett S.D., Brown H.M. (2004). Detection of meat species using TaqMan real-time PCR assays. Meat Sci..

[24] Soares S., Amaral J.S., Oliveira M.B.P., Mafra I. (2013). A SYBR Green real-time PCR assay to detect and quantify pork meat in processed poultry meat products. Meat Sci..

[25] Deng L., Li A., Gao Y., Shen T., Yue H., Miao J., Li R., Yang J. (2020). Detection of the bovine milk adulterated in camel, horse, and goat milk using duplex PCR. Food Anal. Methods.

[26] Giglioti R., Polli H., Azevedo B.T., Katiki L.M., Vercesi Filho A.E. (2022). Detection and quantification of adulteration in milk and dairy products: A novel and sensitive qPCR-based method. Food Chem. Mol. Sci..

[27] Liao J., Liu Y., Yang L., Li F., Sheppard A. (2017). Development of a rapid mitochondrial DNA extraction method for species identification in milk and milk products. J. Dairy Sci..

[28] Sultana S., Hossain M.M., Azlan A., Johan M.R., Chowdhury Z.Z., Ali E. (2020). TaqMan probe based multiplex quantitative PCR assay for determination of bovine, porcine and fish DNA in gelatin admixture, food products and dietary supplements. Food Chem..

[29] Rojas M., González I., Pavón M.Á, Pegels N., Lago A., Hernández P.E., García T., Martín R. (2010). Novel TaqMan real-time polymerase chain reaction assay for verifying the authenticity of meat and commercial meat products from game birds. Food Addit. Contam. Part A.

[30] Ali M.E., Hashim U., Mustafa S., Man Y.B.C. (2012). Swine-specific PCR-RFLP assay targeting mitochondrial cytochrome b gene for semiquantitative detection of pork in commercial meat products. Food Anal. Methods.

[31] Kim M.-J., Kim H.-Y. (2018). Development of a fast duplex real-time PCR assay for simultaneous detection of chicken and pigeon in raw and heat-treated meats. Food Control.

[32] Cheng X., He W., Huang F., Huang M., Zhou G. (2014). Multiplex real-time PCR for the identification and quantification of DNA from duck, pig and chicken in Chinese blood curds. Food Res. Int..

[33] Tichy H.-V., Bruhs A., Palisch A. (2020). Development of real-time polymerase chain reaction systems for the detection of so-called “superfoods” chia and quinoa in commercial food products. J. Agric. Food Chem..

[34] Iwobi A., Sebah D., Kraemer I., Losher C., Fischer G., Busch U., Huber I. (2015). A multiplex real-time PCR method for the quantification of beef and pork fractions in minced meat. Food Chem..

[35] Genis D.O., Bilge G., Sezer B., Durna S., Boyaci I.H. (2019). Identification of cow, buffalo, goat and ewe milk species in fermented dairy products using synchronous fluorescence spectroscopy. Food Chem..

[36] Agrimonti C., Bottari B., Sardaro M.L.S., Marmiroli N. (2019). Application of real-time PCR (qPCR) for characterization of microbial populations and type of milk in dairy food products. Crit. Rev. Food Sci. Nutr..

