# Supplementary material for: Simultaneous Detection of Eight Dairy-Derived Components Using Double-Tube Multiplex qPCR Based TaqMan Probe
Source: Foods. 2024 Oct 10;13(20):3213. doi: 10.3390/foods13203213 (PMC11507643; doi:10.3390/foods13203213)
Supplement: Supplementary file 1 [file foods-13-03213-s001.zip › foods-3245933-supplementary.pdf]

Table S1 Specificity test of multiplex qPCR assay

Animal species tested	Multiplex qPCR system	
	Increase of fluorescence signal	Mean Ct value
Cow	+	20.47±0.50
Buffalo	+	25.07±0.68
Yak	+	22.36±0.16
Goat	+	20.13±0.06
Sheep	+	20.41±0.81
Horse	+	23.46±0.26
Donkey	+	24.21±0.81
Camel	+	24.63±0.93
Soybean	-	-
Corn	-	-
Wheat	-	-
Sweet potatoes	-	-
Rice	-	-

Notes: "+" is positive PCR result (Ct value < 40), and "-"is no increase of the fluorescence signal within 40 cycles.
